# Louisville Medical Institute

**Published:** 1843-04

**Authors:** 


### LOUISVILLE MEDICAL INSTITUTE.

The published Catalogue of this Institution shows that there were one hundred and eighty-nine students in attendance during the past winter. Of these forty-six were from Kentucky, forty-six from Tennessee, twenty-eight from Mississippi, twenty-four from Alabama, thirteen from Ohio, twelve from Indiana, and the remainder from Louisiana, Georgia, the Carolinas, Virginia, Missouri, Illinois, Michigan, &c., &c. There were thirty-seven graduates. The Honorary degree of Doctor of Medicine was conferred upon the following gentlemen: Josiah Higgason, Somerville, Tennessee; Joseph M. Wood, Liberty, Missouri; Rufus Haymond, Brookville, Indiana; Joseph R. Buchanan, Kentucky. C.

